# The Population of the World

**Published:** 1880-11

**Authors:** 


### THE POPULATION OF THE WORLD.

Dr. Behn and Professor Wagner, German geographers of standing, who have devoted much attention to the statistics of population, have just issued a new edition of their calculations. They arrive, after great labor, at results, which we quote, because they modify materially the estimate popularly current in Great Britain:

| | |
|---|---|
| Europe | 315,929,000 |
| Asia | 838,704,000 |
| Africa | 205,679,000 |
| America | 94,495,500 |
| Australia and Polynesia | 4,031,000 |
| Polar regions | 82,000 |
| The world | 1,455,923,500 |

The calculation for Europe, which must be substantially accurate, is much larger than the usual one; but even then the immense bulk of humanity, ten in fourteen of mankind dwells in Asia and Africa.

